# *ϵ*-Machine and *ϵ*-Transducer Analysis of Functional Differentiation in Ant Collectives †

**DOI:** 10.3390/e28070749

**Published:** 2026-07-01

**Authors:** Norihiro Maruyama, Michael Crosscombe, Shigeto Dobata, Takashi Ikegami

**Affiliations:** 1 Department of General Systems Studies, Graduate School of Arts and Sciences, The University of Tokyo, Tokyo 153-8902, Japan; 2 Alternative Machine Inc., Tokyo 150-0001, Japan; 3 Atomi Information Science and Arts Center, Atomi University, Tokyo 112-8687, Japan

**Keywords:** collective behaviour, ant, *ϵ*-machine, *ϵ*-transducer, symbolic dynamics, behavioural differentiation

## Abstract

We investigate functional behavioural differentiation in genetically homogeneous animal collectives using the ϵ-machine and ϵ-transducer frameworks from symbolic dynamics. Long-term tracking of unmarked individuals in colonies of the clonally reproducing ant *Pristomyrmex punctatus* reveals two distinct movement modes—clustering within the group and solitary exploration outside it. Reconstructed individual ϵ-transducers expose a sharp asymmetry in computational structure between these modes: solitary explorers are described by a deterministic machine, whereas clustering ants require stochastic machines to capture their complex patterns of micro-movement. A population-level (universal) ϵ-transducer, inferred from pooled data, captures the shared behavioural repertoire across all individuals. Individual differences are parsimoniously explained as biased and partial traversals of a common state space rather than as distinct generative programs. We compare three predictive models: the ϵ-machine, which relies solely on an ant’s own output history; a memoryful ϵ-transducer, which additionally conditions on changes in the local neighbour count as social input; and a memoryless ϵ-transducer, which uses this social input alone. The memoryful transducer matches the ϵ-machine in prediction accuracy despite requiring ten times as many states, while the memoryless transducer performs substantially worse. This shows that an ant’s own behavioural history is the essential predictor of its future movement at the temporal resolution examined here. We argue, however, that this predictive redundancy does not entail the causal irrelevance of social input: the behavioural history itself accumulates the trace of past social encounters so that any role differentiation established through prior interactions is already inscribed in the output sequence that the ϵ-machine reads, and mode transitions—the moments at which social input most plausibly exerts causal influence—are rare events that contribute negligibly to aggregate one-step accuracy. Agent-based simulations driven by the universal ϵ-transducer reproduce basic motion statistics and transient aggregations but fail to generate the stable macroscopic clusters observed experimentally, pointing to the role of additional mechanisms such as longer-term memory or stigmergic coupling. Nevertheless, ants do respond to their social environment: an explorer encountering an increase in neighbours is absorbed into the cluster and ceases directed movement. Together, our results suggest a two-level organisation: within each behavioural mode, individual dynamics are self-sufficient for one-step prediction, while transitions between modes are environmentally triggered and represent switches between fundamentally different classes of dynamical organisation.

## 1. Introduction

Collective behaviour emerges from local interactions among individuals without central control, and understanding how such macroscopic patterns arise from microscopic behavioural rules remains a central question in ethology, complex systems, and artificial life [1,2,3,4]. Social insects provide a particularly rich model system for studying this problem. Despite consisting of individuals with limited perceptual and cognitive capacities, their colonies often exhibit robust and adaptive collective intelligence [5]. The clonal ant *Pristomyrmex punctatus* is suitable for investigating the origins of colonial intelligence. This queenless species reproduces by obligate thelytokous parthenogenesis [6,7], producing colonies that often consist of a single clonal lineage with high intracolonial relatedness [8]. Yet even within such genetically uniform groups, colonies display pronounced variation in individual movement patterns and task participation. Such behavioural individuality within clonal groups has been documented across social insects [9], including other clonal ant species [10,11], and raises a fundamental question: are these behavioural differences intrinsic properties of individuals, or do they emerge from social interactions and collective dynamics?

Addressing this question requires two key methodological components: the ability to track the behaviour of each individual continuously and to infer their internal states from the observed behaviour. While simulation models can access the internal states of agents directly, experimental studies of living organisms must estimate these states indirectly from behavioural observations. Recent advances in computer vision and machine learning, particularly convolutional neural networks for multi-object tracking, have made it possible to track unmarked individuals over long periods with high spatial and temporal resolution in controlled environments [12]. These developments make it possible to analyse behavioural dynamics at the level of entire colonies. Our previous studies [13] applied such tracking techniques to *P. punctatus* colonies and analysed the resulting trajectories using the ϵ-machine and ϵ-transducer frameworks [14,15,16] from symbolic dynamics. An ϵ-machine is a minimal stochastic finite-state automaton inferred from an output sequence alone, while an ϵ-transducer extends this by conditioning on an input stream, modelling behaviour as an input–output process. Comparing the two allows us to ask whether social input—such as the number of neighbouring individuals—adds predictive power beyond what the ant’s own behavioural history provides.

Across multiple experiments, we observed a consistent differentiation within otherwise homogeneous colonies. Solitary explorers were described by a deterministic machine—they simply persist in motion—whereas clustering ants required multi-state, stochastic machines to capture their complex patterns of micro-movement within the group. To assess the role of social context, we further compared three predictive models: the ϵ-machine, which relies solely on an ant’s own output history; a memoryful ϵ-transducer, which additionally conditions on changes in the local neighbour count as social input; and a memoryless ϵ-transducer, which uses social input alone. The memoryful ϵ-transducer matched the ϵ-machine in prediction accuracy despite requiring far more states, while the memoryless transducer performed substantially worse. This indicates that an ant’s own behavioural history is the essential predictor of its future movement, and that the social input tested here is redundant—its information is already implicit in the ant’s output history (we examine the scope of this predictive redundancy and its distinction from causal irrelevance in Section 4). In longer recordings, we further observed globally synchronised bursting events within clusters, suggesting a link between individual behavioural modes and large-scale temporal coordination.

Here, we introduce a perspective that we refer to as the “community-first” hypothesis [17]. In some studies of animal behaviour, behavioural individuality or personality is often interpreted as arising from intrinsic differences between individuals or from pre-existing traits [9], and classical models attribute division of labour to heterogeneous response thresholds [18,19,20], although recent work has questioned whether thresholds alone suffice [21]. In contrast, the community-first hypothesis proposes that individuality may emerge from the formation of a higher-level entity that we call the community. Under this view, the community is not merely a collection of interacting individuals but an emergent organisational level that can influence the behaviour of its members. As this collective structure develops, it shapes the behavioural tendencies of individuals, leading to the emergence of distinct behavioural roles or personalities. In the case of social insects such as ants, this appears as functional differentiation among otherwise similar individuals [19]. Recent advances in social insect neuroscience further suggest that such behavioural differentiation has identifiable correlates in the ant nervous system, opening the way for a circuit-level account of how collective organisation shapes individual brains [22]. More generally, this perspective suggests that personality may be partly constructed through the dynamics of the community itself rather than arising solely from intrinsic properties of the agents. In this sense, the hypothesis can be viewed as a form of top-down causation operating in collective behavioural systems.

Building on these previous studies and theoretical background, the present study aims to provide a unified account of behavioural differentiation and collective dynamics in *P. punctatus*. Specifically, we seek to (i) characterise individual behavioural modes by reconstructing ϵ-transducers for each ant and by examining the qualitative shift between deterministic and stochastic dynamical regimes that accompanies mode switching, (ii) construct a population-level (universal) model and compare ϵ-machines, memoryful ϵ-transducers, and memoryless ϵ-transducers to assess whether social input adds predictive power beyond an ant’s own behavioural history, and (iii) explore whether behavioural differentiation can be understood as differential usage of a shared behavioural mechanism and test this interpretation through agent-based simulation.

## 2. Methods

### 2.1. Ant Species and Colony Maintenance

We used the ant species *Pristomyrmex punctatus*, a queenless species in which workers reproduce via thelytokous parthenogenesis.

Colonies were collected between 2021 and 2023 from natural populations in Chofu City, Tokyo, in July 2021 (JDJ) and Komaba Campus of the University of Tokyo, Japan, in April 2023 (KA and KB). They were kept in the laboratory under ca. 25 °C. The colonies were maintained separately in a plastic case with test tubes as nesting sites. Each tube was half-filled with water that was cotton-plugged. Artificial Bhatkar diet (slightly modified) or insect jelly (Pro Jelly, KB Farm, Japan) was fed at least twice a week.

**Figure 1:**
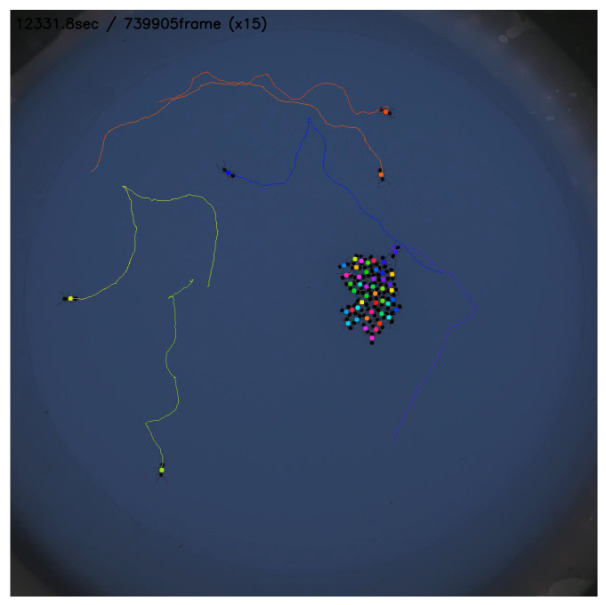
Snapshot of the experimental setup and tracking results. In this example, 50 ants are placed in a circular arena. Each colour trajectory corresponds to one individual.

Behavioural experiments were conducted in shallow, circular acrylic arenas designed to restrict vertical movement while allowing unobstructed horizontal locomotion (Figure 1). The arenas were 100 mm in diameter, with heights of either 1.0 mm or 1.2 mm depending on the trial. Uniform illumination was provided during the experiments.

Two experimental regimes were conducted. In the first, 64 workers from colony JDJ were observed for approximately two hours. In the second, 50 workers from two colonies (KA and KB) were observed for five hours, and the last three hours were used for analysis. All ants used in the experiments were inside-nest workers. This selection was intentional: outside-nest workers tend to include foragers whose behavioural roles may already be differentiated through factors such as age or prior experience, which would confound the present study’s central question of how functional differentiation *emerges* within an initially homogeneous population. Restricting the focal population to inside-nest workers therefore provides a behavioural starting point that is as uniform as possible with respect to known sources of pre-existing role differentiation.

As detailed in the next section, all individuals in the arena were tracked simultaneously, and tracking data from those successfully tracked throughout the recording were used in the analysis.

### 2.2. Tracking and Trajectory Extraction

The arenas were recorded from directly above using a 4K video camera operating at 60 frames per second. Video frames were cropped to arena boundaries and downscaled for efficient processing. For longer recordings, footage was divided into one-hour segments prior to tracking to reduce the propagation of tracking errors.

**Figure 2:**
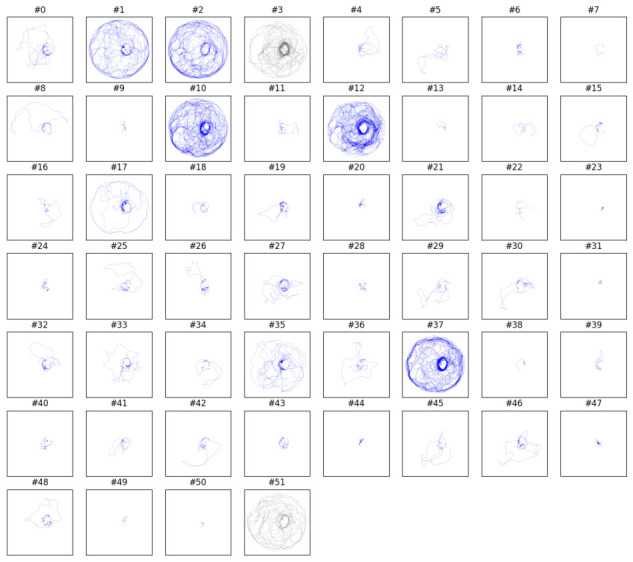
Example of individual trajectories of colony KA. The individuals shown in blue were successfully tracked for the entire duration of the experiment without any tracking loss. Those shown in grey experienced tracking loss during the recording and were therefore excluded from the analysis.

Detection and tracking were performed without physical marking using a convolutional neural network based on the Centroid U-Net architecture, which is a modified version of U-Net, an image segmentation neural network model adapted to object detection [23,24]. A small number of frames from each experiment were manually annotated to provide a training set. Tracking between frames was achieved via nearest-neighbour matching, yielding continuous two-dimensional trajectories for all individuals. Examples of tracking results are shown in Figure 2.

### 2.3. Behavioural Discretisation

To apply the ϵ-machine and ϵ-transducer frameworks, behavioural data must be expressed as discrete symbolic sequences. Our goal in the following procedure was therefore to construct a minimal discretisation that preserved the essential structure of ant behaviour while remaining compatible with reliable state reconstruction.

**Figure 3:**
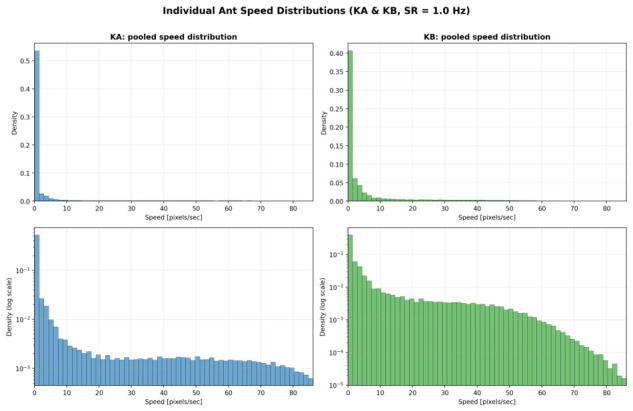
Empirical distribution of instantaneous speeds, pooled across all individuals and time steps in the analysed recording window (colonies KA and KB, 2–5 h, 1 Hz sampling), shown on both linear (**top row**) and log (**bottom row**) scales. Left column: colony KA; right column: colony KB. Both colonies exhibit a sharp peak at v=0—more than an order of magnitude above the density at the first non-zero bin on the log scale—followed by a broad, slowly decaying tail extending to roughly v≈80 pixels/s.

From the trajectories, instantaneous speeds were computed as the Euclidean displacement between consecutive frames. The data were temporally downsampled to a resolution of one second. Each time step was classified as either active or inactive using a binary threshold on instantaneous velocity, set at $v_{\mathrm{thr}}=0$ pixels/s. An individual whose position changed between consecutive 1 Hz sampling steps (v>0) was assigned $Y_{t}=1$; an individual whose position remained unchanged (v=0) was assigned $Y_{t}=0$. This choice is motivated by the empirical distribution of speeds (Figure 3), which exhibits a sharp peak at v=0 standing well above the remainder of the distribution—more than an order of magnitude higher than the density at v≈1 pixel/s—followed by a broad, slowly decaying tail extending to roughly v≈80 pixels/s. This structure indicates a clear qualitative separation between truly stationary individuals at v=0 and a continuum of active locomotion at v>0. The threshold $v_{\mathrm{thr}}=0$ thus isolates the stationary spike from all states involving any detectable movement. No additional hysteresis or minimum-duration constraints were imposed. The robustness of our results to this threshold setting is examined in Appendix C.

The input to an individual (i.e., local social context) was defined based on the number of neighbouring individuals within a fixed radius of 15 pixels from the focal individual at each time step. Neighbours were defined purely by spatial proximity, i.e., whether another individual’s position fell within this radius, without considering orientation or body contact. The neighbour count was then converted into a binary input signal $X_{t}$ representing temporal change in local density: if the number of neighbours increased or decreased (i.e., any change in neighbour count) relative to the previous time step, $X_{t}=1$, whereas if the number remained unchanged, $X_{t}=0$.

Intuitively, one might encode the presence or absence of neighbours as a binary input signal. However, in dense clusters or in their immediate vicinity, such a definition would produce a constant input of $X_{t}=1$ for most time steps, providing little information about the local interaction dynamics. We therefore used temporal changes in neighbour count as the input signal, with the intention of capturing fluctuations in local density within clusters and events in which individuals join or leave near the cluster boundary. Results from auxiliary analyses using a simple neighbour-presence input are provided in Appendix A.

**Figure 4:**
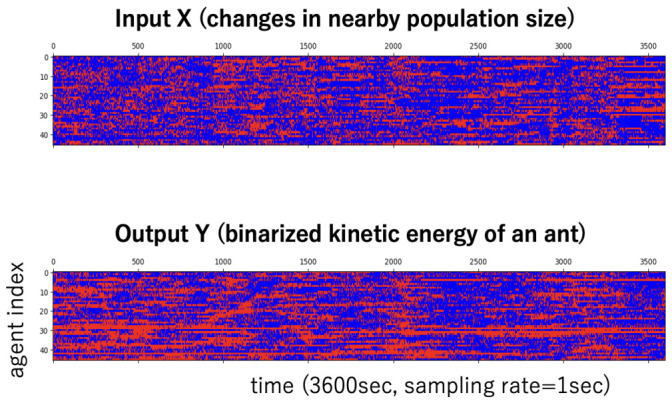
Example of the time series used for reconstruction (colony KA). The input *X* is a binary variable representing whether the number of neighbouring individuals within a fixed radius of the focal ant changed (X=1, red) or remained unchanged (X=0, blue), and the output *Y* is a binary variable representing whether the individual moved (Y=1, red) or remained stationary (Y=0, blue).

Under these definitions, each ant’s behaviour was represented as a discrete-time binary input–output time series (Figure 4).

Note that missing frames in the tracking data were interpolated when possible. However, individuals whose tracks were completely lost during the experiment were excluded from the analysis. As a result, only individuals that were continuously tracked throughout the entire observation period were used in the analysis.

### 2.4. ϵ-Machine and ϵ-Transducer Reconstruction

To reconstruct behavioural dynamics from these time series, we employed the ϵ-machine and ϵ-transducer frameworks from symbolic dynamics.

An ϵ-machine reconstructs a predictive model directly from an observed output time series by identifying the minimal set of internal states required to optimally predict the future of a system from its past [15,25]. The key concept is the *causal state*. Rather than assuming hidden states a priori, causal states are defined by grouping together past histories that lead to the same statistical predictions about the future. More formally, two past histories belong to the same causal state if they induce identical conditional probability distributions over future outputs. Each causal state therefore represents a set of pasts that are predictively equivalent. Transitions between causal states occur as new observations are made, resulting in a stochastic finite-state machine that captures the predictive structure of the process.

An ϵ-transducer generalises this idea to systems that respond to external inputs. Whereas an ϵ-machine predicts future outputs from past outputs alone, an ϵ-transducer reconstructs causal states from past input–output histories and predicts future outputs conditioned on both the current input and the reconstructed state [14]. Comparing the two allows us to assess whether social input (the change in the number of neighbours) adds predictive power beyond what the ant’s own behavioural history provides. We additionally consider a *memoryless* transducer, which conditions predictions on social input alone without access to the ant’s output history, to isolate the contribution of self-generated memory.

Formally, let $X_{t}$ denote the input at time *t*, $Y_{t}$ the output at time *t*, and $s_{t}$ the causal state at time *t*. Let $X_{\mathrm{past}}$ and $Y_{\mathrm{past}}$ denote the histories of inputs and outputs up to time *t*.

- **ϵ-machine**: predicts the next output from the current causal state reconstructed from past outputs,
  $$P\left(Y_{t}|s_{t}\right),\;s_{t}=\epsilon \left(Y_{\mathrm{past}}\right).$$
- **ϵ-transducer**: predicts the next output from the causal state and the current input,
  $$P\left(Y_{t}|s_{t},X_{t}\right),\;s_{t}=\epsilon \left(X_{\mathrm{past}},Y_{\mathrm{past}}\right).$$
- **Memoryless ϵ-transducer**: predicts the next output from the causal state and the current input while ignoring past outputs,
  $$P\left(Y_{t}|s_{t},X_{t}\right),\;s_{t}=\epsilon \left(X_{\mathrm{past}}\right).$$

Reconstruction was carried out using the Causal State Splitting Reconstruction (CSSR) algorithm [26], with history length (i.e., the length of the past histories $X_{\mathrm{past}}$ and $Y_{\mathrm{past}}$) and a complexity penalty as hyperparameters. We searched history lengths up to L=4 and used the maximum value, L=4, in the subsequent analyses, together with a complexity penalty of α=0.001.

Using this reconstruction procedure, we performed three complementary analyses:

- **Individual-Level Reconstruction:** For individual-level analysis, ϵ-transducers were reconstructed separately for each ant using its input–output time series alone. These automata capture individual movement dynamics based on the ant’s own behavioural history.
- **Population-Level (Universal) Reconstruction:** To construct a population-level model, input–output time series from all individuals were pooled, and a single universal ϵ-transducer was reconstructed. This universal ϵ-transducer represents a shared behavioural grammar underlying ant movement, independent of individual identity.
- **Model Comparison:** To assess the role of social input, we compared three models at the population level: (i) the ϵ-machine (output only), (ii) a (memoryful) ϵ-transducer (output and input), and (iii) a memoryless transducer (input only). Models were compared in terms of the number of reconstructed states and prediction accuracy.

### 2.5. Simulation Framework

Agent-based simulations were conducted using the reconstructed universal ϵ-transducer. Each agent followed the transition rules of the shared automaton, emitting movement states as outputs based on its own behavioural history. This allowed for a generative examination of whether autonomous individual dynamics alone can produce collective patterns.

## 3. Results

### 3.1. Behavioural Differentiation in Real Ant Collectives

**Figure 5:**
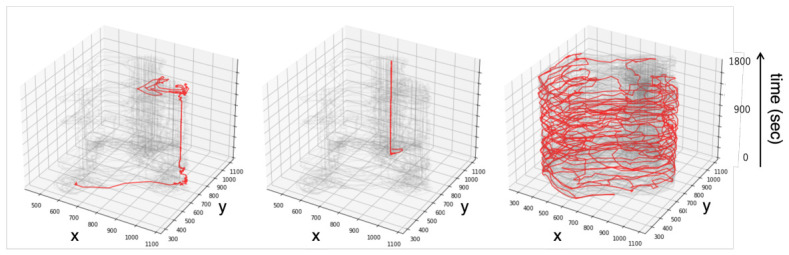
Typical examples of behaviour. Red marks the focal individual; grey shows the trajectories of all other individuals. Each panel shows a different individual over a representative 1800 s interval of the experiment. **Left**: wandering around the colony. **Centre**: “freezing” inside the colony. **Right**: exploring the outside of the colony for a long time.

Tracking revealed pronounced variation in movement patterns within genetically homogeneous colonies. Individuals could be broadly categorised as clustered, wandering, or exploring (Figure 5). These behavioural modes were stable for several minutes to several hours. Across experiments with different colonies, a few individuals still engaged in exploratory behaviour, while many formed a dense cluster. This pattern appears to be a typical collective pattern observed when many individuals of this species are gathered.

**Figure 6:**
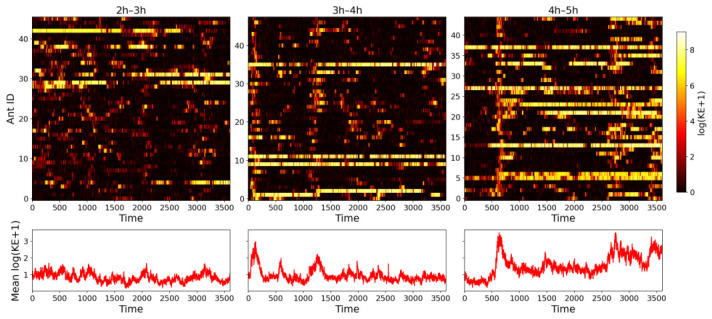
Kinetic energy of each individual (**top**) and the mean kinetic energy (**bottom**) during the last three hours of the five-hour experiment of colony KA (log scale). The first two hours were excluded from the analysis because they contained many transient movements. Note that ant indices do not correspond across the three top panels, since tracking is limited to a maximum of one hour.

At the colony level, in longer recordings, we observed intermittent bursting events in which large numbers of clustered ants became active nearly simultaneously (Figure 6). These events were frequently preceded by the arrival of exploratory individuals at the cluster periphery, suggesting a potential temporal association between exploratory visits and subsequent collective activation.

**Figure 7:**
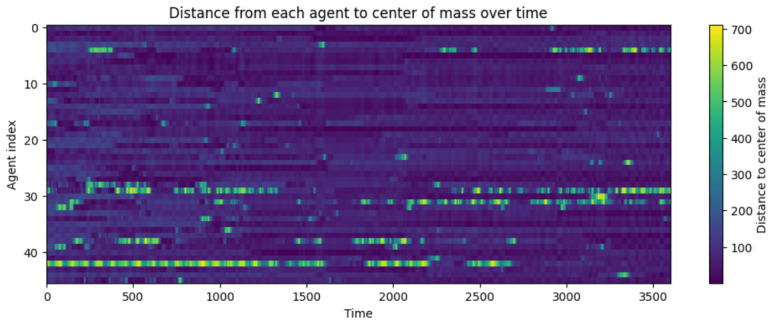
Distance from each individual to the centre of mass of all individuals over time. Because most ants remain within the cluster, this centre nearly coincides with the cluster centre. Each row corresponds to one agent; colour encodes distance (dark: near cluster centre, bright: far from cluster). A few individuals repeatedly exhibit large distances (exploration), while most remain near the centre. Some individuals transition from stationary to exploratory behaviour during the recording. Shown here is part of the experiment on colony KA.

Figure 7 shows the distance from each individual to the colony’s centre of mass over time. Most individuals remain close to the centre (within the cluster), but a few repeatedly venture far from it. Notably, the same individuals tend to re-enter exploration multiple times, suggesting that the propensity to explore is not randomly distributed but biased toward particular individuals over the observed time scale. At the same time, some individuals who were initially stationary begin exploring partway through the recording, indicating that roles are not permanently fixed. Exploratory individuals typically moved continuously at relatively high speeds, resulting in consistently high kinetic energy compared with ants within the cluster, whose movements consisted mainly of small intermittent displacements. This sustained locomotion is reflected in the reconstructed automata as discussed below, where exploratory ants are expected to produce long sequences of Y=1.

### 3.2. Individual ϵ-Transducer

**Figure 8:**
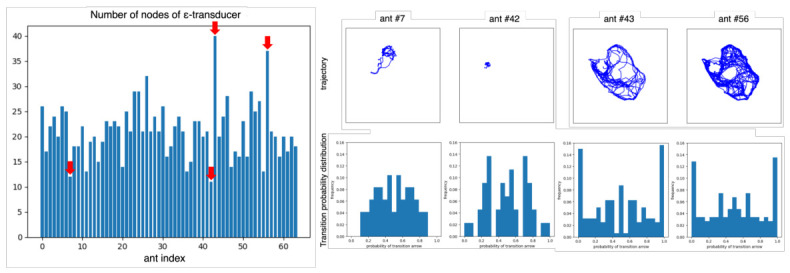
Example of ant behaviour patterns and characteristics of the reconstructed ϵ-transducer. The left shows the number of nodes in the ϵ-transducer for each individual (N=64). The right shows the trajectories of four typical individuals (indicated by red arrows on the left) and the distribution of transition probabilities in their ϵ-transducers.

Reconstructed individual ϵ-transducers revealed a clear correspondence between the behavioural mode described above and the automaton structure. Many automata comprised dozens of nodes, making their overall organisation hard to interpret visually. However, the node counts and transition properties revealed several interesting patterns. For example, clustered individuals were typically described by low-complexity automata with transition probabilities near 0.5, indicating stochastic dynamics. In contrast, exploring individuals exhibited higher-complexity automata with near-deterministic transitions. Figure 8 shows four typical examples of these behaviours and their transition probabilities.

As shown in the figure, a clear contrast can be observed: exploring individuals are characterised by many nodes with deterministic transitions (p≈1 or 0), whereas individuals within clusters exhibit a relatively small number of nodes with transition probabilities close to p=0.5. This mode switch thus corresponds to a qualitative shift from deterministic, patterned dynamics to stochastic, high-entropy dynamics.

**Figure 9:**
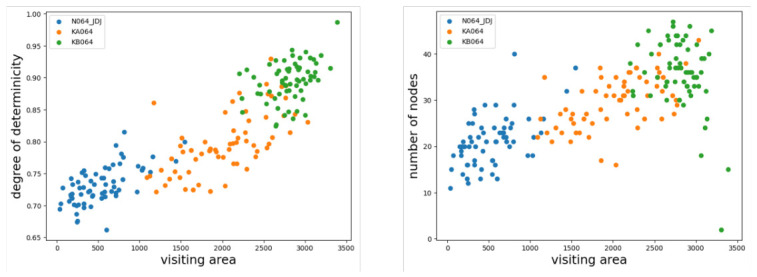
Relationship between the spatial extent of individual behaviour and properties of the reconstructed ϵ-transducer. In both panels, the horizontal axis indicates the total area visited by each individual during the experiment; individuals plotted further to the right traversed larger regions and therefore exhibited more exploratory behaviour. Colours indicate different experiments (colony KA, KB, and JDJ). **Left**: Mean transition probability of each individual’s ϵ-transducer. Higher values (upper positions) indicate dynamics that are closer to deterministic behavioural sequences. **Right**: Number of nodes (causal states) in each reconstructed ϵ-transducer. Individuals plotted higher possess structurally more complex transducers.

Figure 9 verifies this tendency across all individuals and across three experiments (different colonies). Here, we assume that exploratory individuals tend to cover a larger area during the experiment. We therefore compare, for each individual, the area it visited with the degree of determinism (or stochasticity) of its state machine, as well as the complexity of that state machine. These results confirm that the state machines of exploratory individuals tend to be deterministic with a large number of states, while those of clustering individuals tend to be stochastic with a small number of states.

### 3.3. Universal ϵ-Machine/Transducer for Population-Level Modelling

In the previous subsection, we estimated individual-specific ϵ-transducers and, by examining their differences, showed that behavioural characteristics can be analysed using stochastic state machines. However, such an interpretation is close to assuming intrinsic differences among individuals. An important question remains: does individuality depend on internal structure, or is it shaped by interactions within the collective or community? In this section, we instead construct a “universal” ϵ-machine and transducer shared across the population by pooling input–output time series from all individuals. Under this view, all ants possess the same underlying state machine, but individuals may utilise it differently, potentially giving rise to functional differentiation. Additionally, the universal ϵ-transducer enables agent simulations, which will be presented in the following section.

**Figure 10:**
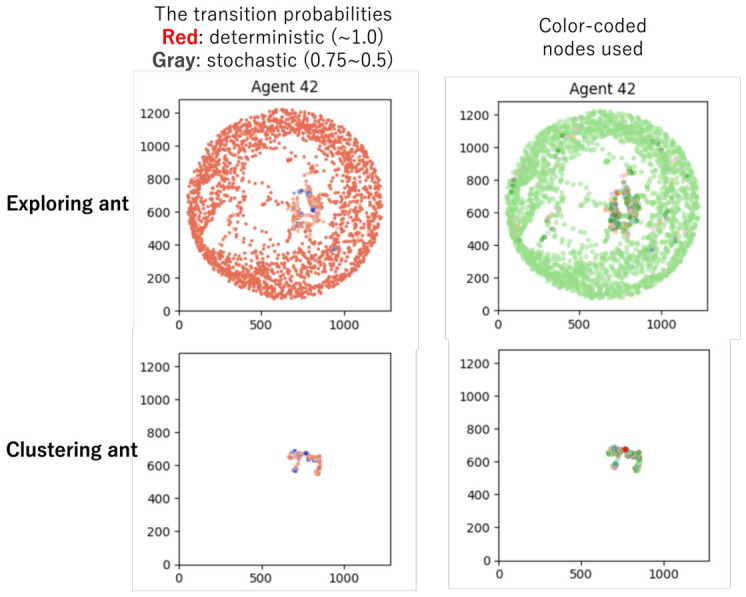
The left panel plots the probabilities associated with the ϵ-transducer transitions used by a given individual during its movement, shown in blue (p=0), grey (p=0.5), and red (p=1.0). The right panel displays the corresponding nodes of the ϵ-transducer, colour-coded according to their identity. The two panels above show individuals exhibiting exploratory behaviour, whereas the two panels below correspond to individuals that remained within the cluster. From the right panel, it can be seen that the exploring individual relies on a single node while traversing the periphery, as indicated by the consistent colour along the trajectory.

We first constructed a population-level ϵ-transducer from pooled input–output time series to examine whether the functional differentiation observed in the individual-level analysis (Section 3.2) is reproduced under a shared model. We then reconstructed an ϵ-machine (from output sequences alone) and a memoryless ϵ-transducer (from input sequences alone) to compare the predictive contributions of individual memory and social input. The reconstructed transducer contained over 100 states, with both deterministic transitions and probabilistic branching. This structure encompasses behavioural patterns shared across all individuals. One individual may frequently transition within a particular subset of this automaton, while another individual may more often transition within a different subset. Figure 10 shows two typical types of individual behaviour and how they utilise the automaton. It visualises which nodes of the automaton were used by the target individual, as well as the probabilities associated with the transitions taken within the automaton related to the trajectory in the arena. Exploratory individuals traverse the entire arena using a single node with deterministic transitions—continuous movement is represented as a self-recursive causal state that persists in outputting Y=1. In contrast, individuals that remain within clusters frequently utilise different nodes and exhibit a higher occurrence of nondeterministic transitions. This pattern is consistent with the results obtained from the individual-specific ϵ-transducers.

**Figure 11:**
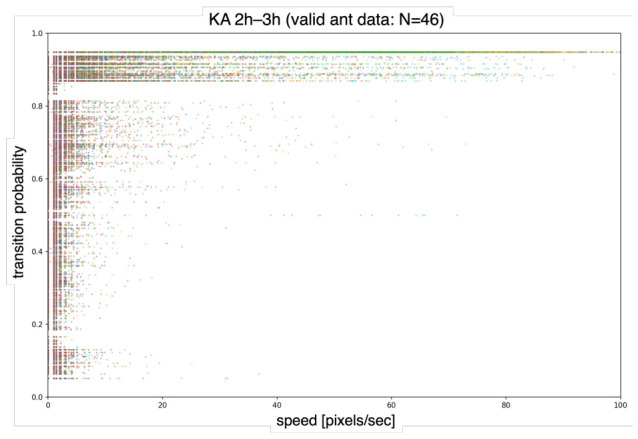
Relationship between instantaneous speed (pixels/s) and the probability associated with the universal ϵ-transducer transition taken at each time step. Each point is a single one-step transition of one individual; colours denote individuals. Stochastic transitions (p≈0.5) appear only at low speed, whereas high-speed movement concentrates on near-deterministic transitions (p≈0.9). Shown here is colony KA, 2–3 h. The counterparts for both colonies and all three recording windows are given in Appendix D.

Figure 11 examines the degree of determinism versus nondeterminism in the transitions as a function of an individual’s instantaneous speed. The vertical axis is the probability that the universal ϵ-transducer assigns to the transition actually taken at each step, i.e., the probability of the emitted output given the causal state and input, $P(Y_{t}|s_{t},X_{t})$; values near 1 correspond to near-deterministic transitions and values near 0.5 to stochastic ones. We take speed as a direct behavioural proxy for mode: sustained high-speed locomotion is the hallmark of exploration, whereas clustering ants stop completely or move slowly and intermittently. The trend is clear and one-directional; at low speed, the transition probabilities span a broad range, from the stochastic transitions (p≈0.5) characteristic of within-cluster micro-movement up to near-deterministic values; stochastic transitions are confined to this low-speed regime, and as speed increases, the points concentrate ever more tightly on the near-deterministic self-recursive state (p≈0.9) that persists in continuous motion. Because the universal ϵ-transducer sees the movement output only in binarised (moving/stationary) form and is therefore blind to the magnitude of speed, this association is an empirical property of the behaviour rather than a consequence of the discretisation: the reconstruction could in principle have assigned fast and slow movement to equally stochastic states, yet the data place faster motion on progressively more deterministic dynamics.

Overall, although all ants share the same automaton, individuals differ in how they traverse it. Note that the individual-level ϵ-transducers reconstructed in the previous subsection assigned many nodes to exploratory individuals, but their transitions were almost entirely deterministic; when data from all individuals are pooled, this regularity is likely collapsed into a single self-recursive causal state in the universal model. Exploring individuals thus occupy a single deterministic state in the shared automaton, whereas clustered individuals traverse broader regions with stochastic transitions. Thus, behavioural differentiation emerges as biased and partial usage of a shared state space.

A key question arises: does social input (the change in the number of neighbouring individuals) add predictive power beyond what the ant’s own behavioural history provides? To answer this, we compared three predictive models (see Section 2.4) reconstructed from the same dataset: an ϵ-machine, a (memoryful) ϵ-transducer, and a memoryless ϵ-transducer.

**Figure 12:**
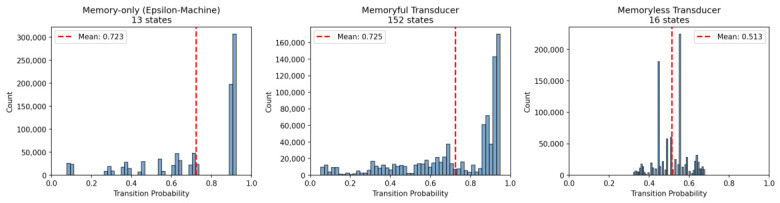
Distribution of transition probabilities used for prediction in the reconstructed models. In the memoryless ϵ-transducer, most transitions are highly stochastic, with probabilities concentrated around p≈0.4–0.6. In contrast, the ϵ-machine shows a distribution closer to that of the full ϵ-transducer, although transitions with probabilities around p≈0.8 are largely absent.

The results are shown in Table 1 and Figure 12. Because the reconstructed ϵ-machine and ϵ-transducer contain many nodes, their full structures are difficult to interpret directly. They are therefore presented in Appendix E. The ϵ-machine (13 states) and the memoryful ϵ-transducer (152 states) achieve nearly identical prediction accuracy, despite the transducer requiring more than ten times as many states. This demonstrates that social input adds no predictive information beyond what is already captured by the ant’s own behavioural history. The memoryless transducer (16 states), which uses neighbour count information alone without output history, performs substantially worse, confirming that self-generated memory—the ant’s own past behaviour—is the essential predictor.

**Figure A6:**
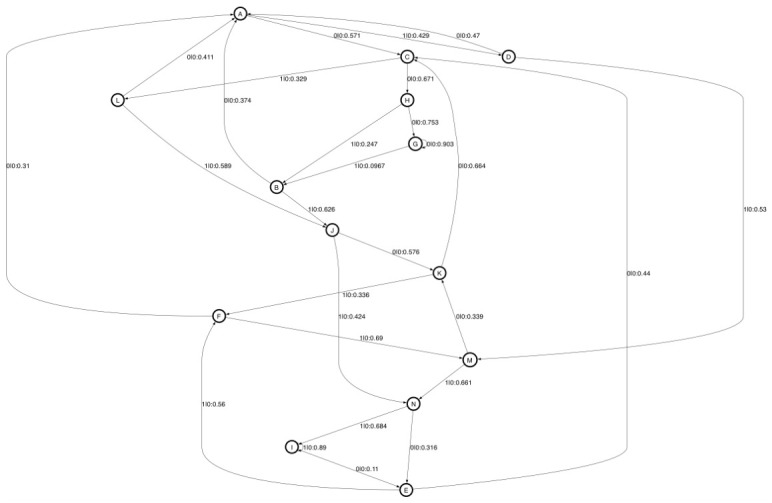
This is an example of an ϵ-machine reconstructed from the data. A self-recursive node (I) that outputs 1 with high probability is associated with exploratory behaviour outside the cluster. In contrast, another node (G) that outputs 0 with high probability may correspond to individuals located within the centre of the cluster.

Within the reconstructed ϵ-machine, one node with a self-recursive transition that emits Y=1 with high probability corresponds to exploratory behaviour (Figure A6): a single deterministic state that persists in motion. The other nodes correspond to the stochastic dynamics observed during clustering. The proliferation of states in the memoryful transducer (from 13 to 152) without predictive gain indicates that conditioning on neighbour count fragments the state space unnecessarily; the social context information is already implicit in the ant’s behavioural history. This result was robust to the choice of input encoding: using the presence of neighbours as input rather than the change in neighbour count reduced the number of transducer states (from 152 to 93), likely because the absolute count remains relatively stable within clusters, but prediction accuracy remained unchanged (Appendix A).

We additionally verified the robustness of the redundancy result to the choice of temporal resolution by re-running the reconstruction at 0.5 s and 2 s sampling (Appendix B). The qualitative pattern—memoryful transducer matching memory-only accuracy while requiring far more states, and memoryless transducer performing substantially worse—holds across all tested resolutions, though absolute state counts vary as expected.

### 3.4. Agent-Based Simulations by the Universal ϵ-Transducer

**Figure 13:**
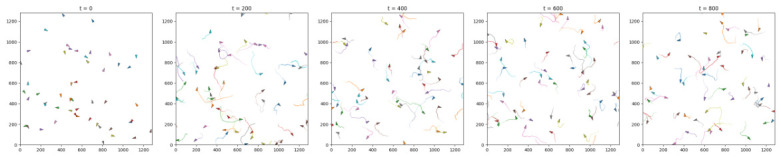
Agent-based simulation results using the universal ϵ-transducer. Time is shown in seconds, and trajectories represent a 120-s interval. Snapshots at t=0,200,400,600, and 800 s from left to right. Some individuals exhibit long-range exploratory movement, while others remain relatively stopped. At certain moments (e.g., t=200 and t=400), transient groupings of agents can be observed; however, these aggregations are short-lived and subsequently disperse.

By constructing a universal ϵ-transducer, it becomes possible to build an agent-based simulation by instantiating multiple copies of the model. Conducting agent-based simulations with interactions was one of the motivations for extending the model from an ϵ-machine to an ϵ-transducer. Because the ϵ-transducer constructed here outputs only movement or stopping, the direction and speed of motion were supplemented by sampling from the empirical distributions obtained from the data. The number of individuals, the size of the simulation area, and the interaction range were set to be the same as those used in the real ant experiments and in the ϵ-transducer reconstruction (for simplicity, periodic boundary conditions were applied). Simulations driven by the universal ϵ-transducer reproduced basic movement patterns (Figure 13).

Simulations exhibited sudden wandering behaviour reminiscent of exploration, and occasionally formed small groupings of a few individuals that could serve as seeds for cluster formation. However, the simulation also shows limitations of the autonomous ϵ-transducer model; large and stable aggregations were difficult to reproduce. These results suggest that individual autonomous dynamics alone—even when faithfully reconstructed from data—are insufficient to sustain large-scale collective organisation. The formation of persistent clusters likely depends on additional mechanisms operating over longer temporal or spatial scales, such as longer-term memory, spatial constraints, or stigmergic coupling.

## 4. Discussion

As introduced in Section 1, our study is grounded in the “community-first” hypothesis [17]. This hypothesis proposes that individuality or personality does not necessarily originate from intrinsic differences among individuals. Instead, it suggests that a community-level organisation first emerges through interactions among individuals, and this emergent community subsequently shapes the behavioural tendencies of its members. (In the case of *P. punctatus*, the dense cluster may represent such a community.) In this view, the community acts as a higher-level entity whose dynamics influence and stabilise individual behavioural patterns. In social insects such as ants, this process appears as functional differentiation among otherwise similar individuals.

Based on this view, this study addresses the question of how functional differentiation or individuality arises and is maintained within a population of homogeneous individuals. More generally, understanding how collective dynamics influence the emergence of individual behavioural differentiation is a key challenge. Our integrated series of experiments provides a possible interpretation of how movement differentiation emerges and persists within colonies of the clonal ant *Pristomyrmex punctatus*. Despite genetic homogeneity and similar initial conditions, individuals consistently segregated into distinct behavioural modes, ranging from stationary clustering to sustained exploration. These modes were stable over several hours, suggesting that they reflect more than transient fluctuations in activity. This parallels recent findings in other clonal ant species, where behavioural individuality emerges without genetic variation and has measurable consequences for colony function [10]. Similar forms of functional differentiation have also been studied through agent-based simulations and theoretical models of collective behaviour [19,20,27], where such differentiation emerges as a collective phenomenon even in homogeneous populations.

### 4.1. Individual-Level Dynamics: Determinism and Stochasticity

Using individual-level ϵ-transducer reconstructions, we found a clear correspondence between behavioural mode and the structure of the inferred automata. Clustered individuals were typically described by relatively low-complexity machines with transition probabilities near 0.5, indicating highly stochastic dynamics with weak dependence on recent history. In contrast, exploring individuals generated automata with larger state repertoires and transitions concentrated near 0 or 1, consistent with deterministic and repetitive movement patterns. The mode switch between exploration and clustering thus corresponds to a qualitative shift in computational regime: from deterministic, many-state dynamics with higher structural complexity to stochastic, high-entropy dynamics. This deterministic–stochastic dichotomy remained robust across multiple reconstruction parameterisations, supporting the interpretation that it reflects a fundamental organisational feature of the colony rather than an artefact of model selection. Additionally, this dichotomy is independently corroborated at the level of instantaneous movement (Figure 11): faster motion maps onto progressively more deterministic transitions even though the reconstruction never sees speed magnitude, so the correspondence between exploratory (fast) movement and deterministic dynamics is not an artefact of how the symbolic sequence was constructed. A similar hierarchical structure in individual behaviour has been reported using information-theoretic methods in other organisms [28], suggesting that the deterministic–stochastic distinction may be a general feature of behavioural differentiation.

### 4.2. Collective Dynamics: Bursts and Colony-Wide Coordination

As described in Section 3.1, in the longer recordings we observed intermittent bursting events in which a large fraction of clustered individuals became active nearly simultaneously before returning to inactivity. These events appeared as discrete episodes of synchronous activation and are reminiscent of collective excitations reported in other social systems [29,30,31]. Qualitative observation suggests a possible temporal association between exploratory visits to clusters and the onset of bursts. While not every visit appeared to trigger a burst, the repeated proximity of explorers to burst initiation suggests that highly mobile individuals may sometimes contribute to colony-wide transitions, for example, by perturbing local contact structure within clusters. Future work should quantify this relationship using appropriate statistical controls.

### 4.3. From Individual-Specific Models to a Shared Behavioural Grammar

In Section 3.2 of this work and our previous work [13], individual behavioural characteristics were analysed by reconstructing each individual’s ϵ-transducer and examining the properties of the resulting automaton. However, a limitation of purely individual-specific reconstructions is that they can be read as implicitly assuming intrinsic differences in internal mechanisms between ants. To address this limitation, we complemented the individual-level analysis with a population-level reconstruction including a universal ϵ-transducer and the corresponding ϵ-machine, inferred from time series pooled across all individuals.

This universal model provides a compact description of a shared behavioural grammar underlying ant movement, while allowing individual differences to emerge through differential usage. Although the automaton structure is common, individuals traverse distinct regions of the state space with different transition biases. Exploring ants occupy a single, deterministic state that persists in motion, whereas clustered ants traverse broader regions with stochastic branching among the remaining states. From this perspective, behavioural differentiation is not primarily encoded in distinct internal “machines” for each ant; rather, it arises as biased and partial traversal of a shared state space. Note that this analysis is restricted to inside-nest workers as an initially homogeneous population; outside-nest workers may carry intrinsic differences (e.g., from age-related polyethism) and could exhibit behaviours beyond the present shared state space.

### 4.4. Predictive Redundancy and the Internalisation of Social Input

The comparison between the ϵ-machine, memoryful ϵ-transducer, and memoryless ϵ-transducer (Section 3.3) clarifies the respective contributions of individual memory and social input. The ϵ-machine (13 states) achieves the same prediction accuracy as the memoryful ϵ-transducer (152 states), which additionally conditions on changes in the number of neighbouring individuals. Meanwhile, the memoryless transducer (16 states), which uses social input alone without behavioural history, performs substantially worse. At first glance, this demonstrates that an ant’s own output history—its memory—is the essential predictor of future movement, and that social input is redundant: whatever information changes in the neighbour count carry is already implicit in the ant’s behavioural history. The proliferation of states in the memoryful transducer without predictive gain reinforces this reading, indicating that conditioning on social input fragments the state space unnecessarily rather than revealing additional meaningful structure. This finding contrasts with information-theoretic studies that have detected significant directed information flow in other collective systems [32,33], suggesting that in *P. punctatus* the coupling between individuals operates on time scales or through channels not captured by instantaneous neighbour count.

The interpretation of this redundancy, however, requires care, and we develop it in detail given its centrality to the present study’s conclusions. Predictive sufficiency and causal irrelevance are distinct properties: a variable can be redundant for one-step-ahead prediction while remaining essential to how the predictor itself came to take its current form. Below we describe three mechanisms—not mutually exclusive—by which social input could be causally important even when it is predictively redundant. They differ in *where* the causal influence is located: in the accumulated history that the ϵ-machine reads (internalisation), in the rare transition events that aggregate accuracy averages over (transition rarity), or in the construction of the analytical units themselves (mode definition).

- **Internalisation of social influence into behavioural history:** The behavioural history $Y_{\mathrm{past}}$ that the ϵ-machine reads is itself the cumulative product of past interactions with nestmates. If a sequence of prior neighbour encounters has driven a focal ant into a clustering mode, the resulting low-energy, stochastic movement pattern is already embedded in $Y_{\mathrm{past}}$, and one-step prediction can proceed from $Y_{\mathrm{past}}$ alone. The ϵ-machine is then not bypassing social input but reading its accumulated trace. Under this interpretation, the equal predictive accuracy of the ϵ-machine and the memoryful ϵ-transducer reflects an *internalisation* of social influence into the behavioural sequence rather than a true independence between behaviour and social context.
- **Rarity of causally decisive events:** Mode transitions—the moments at which social input most plausibly exerts causal influence—are rare relative to the long stretches of within-mode steady state that dominate the time series. With one-second temporal resolution and a binary movement output, the few transition events at which neighbour count may be decisive contribute negligibly to aggregate accuracy. The reported prediction accuracy is therefore dominated by within-mode (clustering or exploring) prediction, and the very small accuracy gain of the memoryful transducer may itself reflect this transition-prediction contribution rather than statistical noise.
- **Mode definitions inherit autonomy from the output sequence:** The two behavioural modes are themselves operationally defined within the same symbolic sequence the ϵ-machine reconstructs: they were extracted as regions of stable output behaviour. Within-mode dynamics therefore appear autonomous in part by construction. This is consistent with—indeed, predicts—that social input acts primarily at the boundary between modes rather than continuously within them. Our direct observations agree with this picture: an explorer encountering an increase in neighbours is absorbed into the cluster and ceases directed motion. This absorption is, by inspection, a causal effect of social input on individual behaviour; it does not improve aggregate prediction accuracy because once the transition has occurred, the resulting clustering trajectory is itself fully predictive of subsequent stationary behaviour without further reference to neighbour changes.

These considerations align with—rather than contradict—the community-first framework that motivates this study. The hypothesis posits that the community (here, the cluster) emerges from interactions and subsequently shapes member behaviour. A causal role for social input that operates predominantly at mode entry and is then carried forward by the individual’s autonomous dynamics is precisely the kind of top-down influence the hypothesis describes. Predictive redundancy of instantaneous neighbour count does not preclude such a causal role.

The scope of the redundancy result should therefore be stated precisely. It applies to the *instantaneous change in neighbour count* for predicting *within-mode movement* at *one-second resolution*, under a *binary discretisation* of both movement and social context. The same qualitative pattern holds under an alternative input encoding (Appendix A), at sampling rates of 0.5 and 2 Hz (Appendix B), and under stricter movement-binarisation thresholds (Appendix C), confirming robustness within the tested range, but these robustness checks do not extend to the longer timescales (minutes to hours) over which social influence on mode composition most plausibly operates. The redundancy result thus leaves open—and our mode-transition observations actively support—a causal role for social input that operates (i) on slower or faster timescales than the present model resolves, (ii) at specific transition events rather than continuously, or (iii) through richer features of the social environment (spatial configuration, body contact, chemical cues) than our binary input encodes. Adjudicating among these possibilities will require interventional manipulations such as the selective introduction or removal of neighbours at controlled moments, which the present observational design cannot provide.

Taken together, these considerations point to a two-level organisation of behavioural dynamics. During exploration, behaviour is autonomous and deterministic, captured by the ϵ-machine as a single self-recursive causal state. Within clusters, dynamics are stochastic and multi-state but still self-determined: social input does not improve one-step within-mode prediction. Transitions between these regimes—mode switches triggered by changes in the social environment—are not captured within either framework and likely reflect dynamics operating at the community level, with the dense cluster as a candidate realisation of such a community. This interpretation is further supported by the agent-based simulation results discussed in the next section.

### 4.5. Simulation with the Universal ϵ-Transducer: Successes and Gaps

Based on the above analysis, we conducted agent-based simulations using the reconstructed universal ϵ-transducer and examined the resulting behaviour (Section 3.4). Agent-based simulations driven by the universal ϵ-transducer reproduced basic movement patterns and generated small, transient clusters. However, large and stable aggregations comparable to those observed experimentally were difficult to reproduce. This gap is informative: it suggests that the universal machine, which captures autonomous individual dynamics, is not sufficient to account for long-term collective structure.

Several non-exclusive mechanisms may be missing from the present generative model. First, longer-term memory or slow internal variables may influence movement decisions beyond the history lengths effectively captured here [34]. Second, spatial constraints and environmental heterogeneity (including boundary effects or persistent spatial cues) may stabilise aggregation [12]. Third, stigmergic effects or chemical communication could provide a longer-lived coupling between individuals than instantaneous neighbourhood changes [35]. Future work integrating such mechanisms into the input representation, or augmenting the transducer framework to incorporate multi-timescale state variables, may clarify what is required for stable large-scale aggregation.

### 4.6. Beyond the Universal Machine: Toward a Continuous-Medium Description of the Cluster

We present here a set of qualitative observations and an interpretive framework that follows from them. A quantitative characterisation of the phenomena described below—propagation speed of activation waves, spatial structure of burst events, and statistical relationship between the two—is beyond the scope of the present study and is identified as an important direction for follow-up work.

We propose that bursts and stable aggregation reflect a level of organisation that the individual-and-input formulation does not appear to capture. Several features of our observations support this proposal. First, burst events involve near-simultaneous activation of individuals distributed across the spatial extent of the cluster, including pairs of ants whose local neighbourhoods do not overlap; such non-local synchrony is difficult to account for through purely local interactions of the form our input encodes. Second, and more strikingly, we observe qualitative indications that activation can propagate through the cluster in wave-like patterns: localised activity in one region of the cluster appears to spread to neighbouring regions with an apparent propagation speed and waveform, recruiting individuals into the active state along its path. Even at this qualitative level, the existence of such propagating waves is suggestive: it points to coherent spatiotemporal dynamics with properties—an apparent propagation speed, a wavefront structure—that are not properties of any individual ant and would not be naturally defined on individual behavioural histories alone. These are, by their nature, the kind of properties associated with a *continuous medium*. Third, the universal ϵ-machine, which captures within-mode individual behaviour with high accuracy, predicts neither the timing of bursts nor the spatial structure of these waves. The agent-based simulation driven by the universal machine likewise fails to produce either stable clusters or wave-like activation patterns. This is consistent with the possibility that the relevant dynamics belong to a different level of description. Fourth, our direct observation of explorer absorption (Section 4.4) shows that the cluster exerts a discrete causal influence on individual mode at the moment of contact: the explorer’s autonomous deterministic dynamics are overridden upon entry into the cluster. Taken together, these observations suggest that the cluster behaves as a continuous medium with its own macroscopic state variables—candidates include local activity fields, density fields, and the spatial structure of contact networks—whose dynamics are not reducible to the aggregate of individual behavioural histories. Under this interpretation, the cluster is not merely a spatial aggregation of independently moving ants but a higher-order dynamical unit whose macroscopic state both supports the propagation of activation waves and shapes the behavioural mode of its constituent individuals. This aligns with the form of top-down causation suggested by the community-first hypothesis (Section 1): the community is not derivative of the individuals composing it but constitutes a distinct level of organisation with its own dynamics, which in turn shapes the individuals at moments of collective transition. A natural formalisation of this picture would describe the cluster by continuum fields satisfying reaction-diffusion-like dynamics, coupled to the individual-level ϵ-machines at points of contact between the two levels. Constructing such a coupled description is beyond the scope of the present study, but our results indicate the potential value of such a formalism and the directions in which it should be developed. What the present analysis establishes is the substantive negative claim that an individual-and-local-input formulation, however refined within its own terms, leaves a structured residual—wave propagation, stable aggregation, synchronous bursting—that corresponds precisely to the collective phenomena the community-first hypothesis predicts.

### 4.7. Implications and Future Directions

Taken together, the individual-level and population-level analyses suggest one possible unified picture of behavioural differentiation in ant collectives. The switch between exploration and clustering is not merely a change in activity level but a transition between fundamentally different computational regimes—deterministic and stochastic—both of which are autonomous with respect to immediate social input within each mode.

However, our conclusion that social input is redundant within behavioural modes may partly reflect the coarseness of the input encoding. As shown in Section 2.3, the binary output encoding $Y_{t}\in \left\{0,1\right\}$ is well supported by the speed distribution itself, which shows a sharp peak at zero with a smoothly decaying tail for positive values; this structure indicates that the stationary/moving distinction reflects a natural feature of the data rather than an arbitrary threshold. A finer discretisation distinguishing, for example, slow and fast-moving states would require placing further thresholds within the broad moving-state mode, where no comparable structural gap exists; such partitions would not correspond to features of the data and would risk introducing artefacts of the encoding choice into the reconstructed dynamics. The binary encoding thus extracts the one distinction that the distribution naturally supports. On the other hand, the binary *input* encoding, by contrast, is a stronger simplification. The present binary input signal discards potentially informative features such as the magnitude of density changes, spatial configuration of neighbours, and physical contact. Future work should explore richer input representations to determine whether this redundancy is robust or an artefact of information loss during discretisation.

A second scope limitation concerns the focal population itself. The present analysis is restricted to inside-nest workers, whose behavioural repertoire is narrower than that of outside-nest foragers. This is consistent with our research question—we examine how differentiation arises in a population that is as uniform as possible with respect to pre-existing roles—but it also means that the reconstructed universal ϵ-machine does not characterise the full behavioural range of *P. punctatus*. An interesting extension would be to apply the same framework to mixed populations, including foragers, and to ask how the shared state space expands or restructures when individuals with already-established roles are added.

Several open questions follow naturally. First, the causal pathways linking exploratory behaviour to collective bursting events require experimental manipulation (e.g., selective removal or constraint of explorers) to establish necessity and sufficiency. Second, the stability of behavioural modes over longer timescales remains to be determined; roles that are stable over hours may still be fluid across days. Finally, identifying the mechanism by which mode switches are triggered—given that social input is redundant for within-mode prediction but environmental changes clearly drive transitions between modes—remains a key challenge for future work.

## 5. Conclusions

In this study, we analysed collective behaviour in the clonally reproducing ant *Pristomyrmex punctatus* by reconstructing behavioural dynamics using both ϵ-machines and ϵ-transducers from the symbolic-dynamics framework. Individual-level reconstructions revealed a sharp differentiation: solitary explorers are described by near-deterministic machines, whereas clustering ants are described by highly stochastic ones. A similar pattern was also observed in how individual ants differ in their use of the population-level universal ϵ-transducer. The universal model is consistent with an interpretation in which behavioural differentiation emerges through differential usage of a shared behavioural grammar rather than through intrinsic differences in internal mechanisms. At the population level, a comparison of three predictive models—the ϵ-machine, ϵ-transducer, and memoryless ϵ-transducer—demonstrated that an ant’s own behavioural history is the essential predictor of its movement, with social input (change in neighbour count) adding no further predictability for one-step prediction within the behavioural modes, although it may play a causal role in both the initial determination of an individual’s mode and transitions between modes. Nevertheless, transitions between exploration and clustering are environmentally triggered, representing switches between deterministic and stochastic computational regimes.

Agent-based simulations driven by the universal model reproduced basic movement patterns and small transient clusters but failed to generate large stable aggregations, indicating that additional mechanisms such as longer-term memory, spatial constraints, or stigmergic coupling are likely required for macroscopic organisation.

In summary, the computational mechanics framework—combining ϵ-machines, ϵ-transducers, their comparison, and the resulting agent-based simulations—provides a principled way to disentangle autonomous individual dynamics from socially driven processes, and offers new perspectives on how behavioural differentiation emerges (e.g., the differentiation between deterministic exploratory and stochastic clustering dynamics) and how individuals switch between modes in homogeneous biological collectives. By testing what generative models built from these reconstructions can and cannot reproduce, this approach offers a compact, interpretable bridge between individual behavioural dynamics, collective organisation, and generative modelling.

## Figures and Tables

**Table 1:** Predictive performance and model complexity of the three reconstructed models.

Model	States	Mean Accuracy	SD
ϵ-machine (memory-only)	13	0.8027	0.0626
Memoryful ϵ-transducer	152	0.8037	0.0627
Memoryless ϵ-transducer	16	0.5686	0.2074

**Figure A1:**
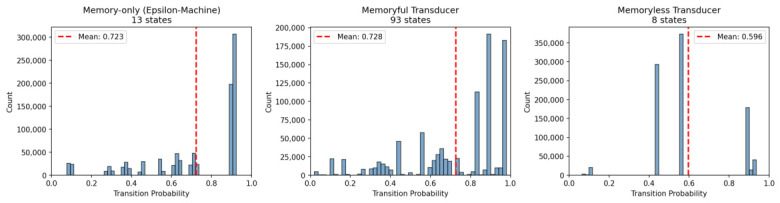
Transition probabilities used by the ϵ-machine and ϵ-transducer when the input is defined as the presence or absence of neighbours (corresponding to Figure 12 in the main text).

**Figure A2:**
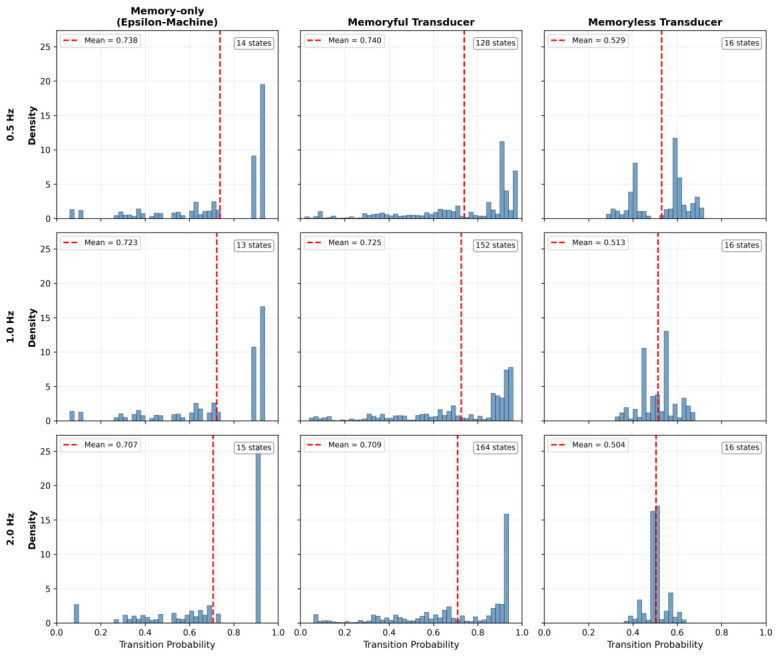
Distribution of transition probabilities for the three reconstructed models at each sampling rate (rows: 0.5, 1.0, 2.0 Hz; columns: memory-only ϵ-machine, memoryful ϵ-transducer, memoryless ϵ-transducer). Red dashed lines indicate the mean transition probability; the number of inferred causal states is annotated in each panel. The overall shape of the distribution—a high-probability mode near p≈0.9 accompanied by a broader stochastic component—is preserved across all rates for both memory-based models, while the memoryless transducer remains concentrated around p≈0.4–0.6 throughout. This is the per-rate counterpart of Figure 12 in the main text.

**Figure A3:**
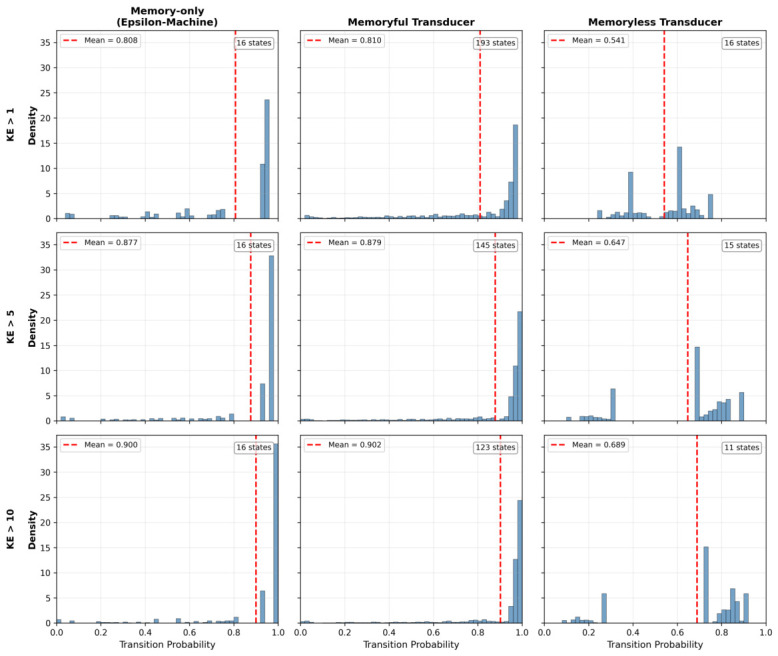
Transition-probability distributions under stricter movement-binarisation thresholds. Rows: a step is coded as moving (Y=1) only when its kinetic energy exceeds KE>1,5,10 (top to bottom). Columns: memory-only ϵ-machine, memoryful ϵ-transducer, and memoryless ϵ-transducer (as in Figure 12). At every threshold, the memory-only and memoryful distributions share the same structure—a broad spread of stochastic transitions together with a pronounced peak at near-deterministic transitions—whereas the memoryless distribution lacks this deterministic peak and stays concentrated at intermediate probabilities, reproducing the main-text result. Raising the threshold shifts all distributions toward higher probabilities as fine-scale fluctuations are removed, without eliminating the stochastic component.

**Figure A4:**
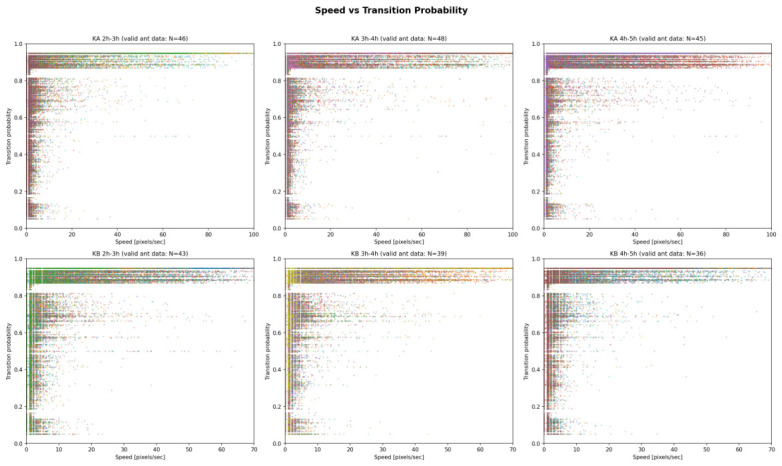
Instantaneous speed versus transition probability of the universal ϵ-transducer transition taken at each time step, for both colonies and all three recording windows. Rows: colonies KA and KB; columns: 2–3, 3–4, and 4–5 h. Axes and colour coding as in Figure 11.

**Figure A5:**
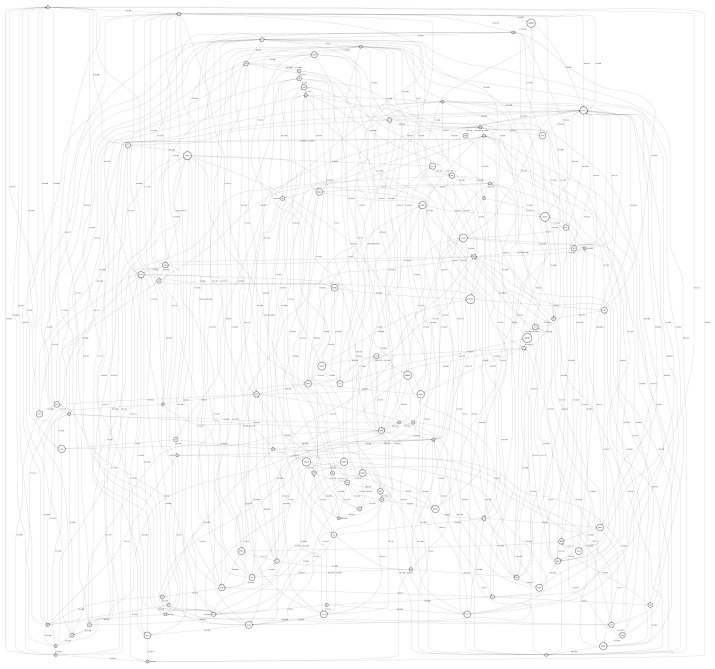
Example of an ϵ-transducer reconstructed from the data. Because it contains approximately 100 nodes, the detailed structure cannot be clearly resolved in the figure.

**Figure A7:**
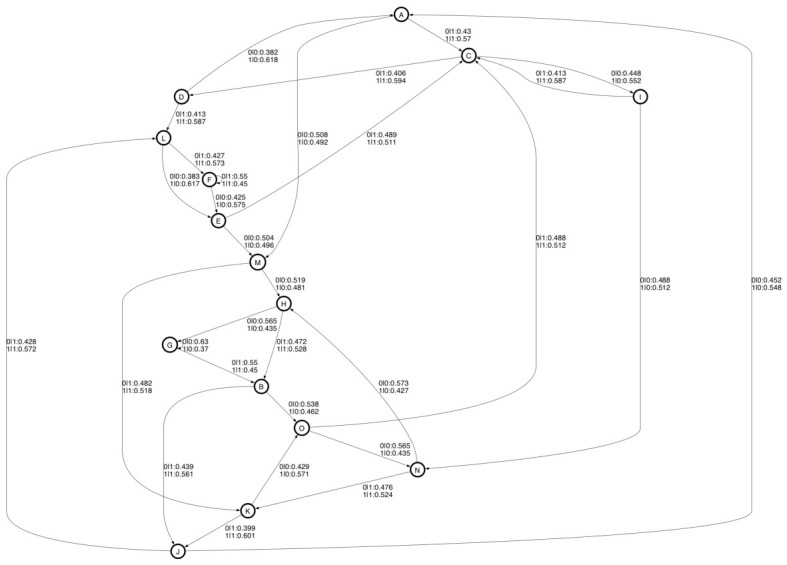
This is an example of a memoryless ϵ-transducer reconstructed from the data. Most nodes exhibit stochastic transitions with probabilities around p=0.4–0.6.

## Data Availability

The data presented in this study are available on request from the corresponding author.

## Appendix A. Alternative Input Definition Based on Neighbour Presence

In the main analysis, the input to the ϵ-transducer was defined using temporal changes in neighbour count rather than simple neighbour presence. As discussed in the Methods section, a binary encoding of neighbour presence ($X_{t}=0/1$) would produce nearly constant input values within dense clusters and their immediate surroundings, providing little variation for the model.

To examine the effect of this choice, we also reconstructed the models using an alternative input definition in which $X_{t}$ indicates only the presence ($X_{t}=1$) or absence ($X_{t}=0$) of neighbouring individuals within the same interaction radius. The resulting model statistics are summarised in Table A1. Note that the ϵ-machine is unaffected by this change, since it does not use the input signal; therefore, its results are identical to those reported in the main analysis. Compared with the results reported in Table 1 in the main text, the prediction accuracy remains nearly unchanged. However, the structural complexity of the reconstructed ϵ-transducer differs substantially from that of the model used in the main analysis.

Figure A1 shows the distribution of transition probabilities used for prediction under this input definition. For the ϵ-transducer, the overall structure of the distribution remains similar, but transitions around p≈0.8 largely disappear, leading to a clearer separation between highly stochastic and nearly deterministic transitions. A similar tendency is observed for the memoryless ϵ-transducer, where the distribution is dominated by two prominent transitions and the surrounding intermediate probabilities largely disappear.

These differences suggest that the simple neighbour-presence input fails to capture certain aspects of local interaction dynamics, particularly fluctuations in density near cluster boundaries or events in which individuals join or leave clusters.

**Table A1:** Predictive performance and model complexity of the three reconstructed models with simple neighbour-presence input (corresponding to Table 1 in the main text).

Model	States	Mean Accuracy	SD
ϵ-machine (memory-only)	13	0.8027	0.0626
Memoryful ϵ-transducer	93	0.8040	0.0636
Memoryless ϵ-transducer	8	0.6556	0.1078

## Appendix B. Effect of Temporal Resolution

To assess whether the conclusions depend on the temporal resolution of the discretized trajectories, we repeated the analysis at three sampling rates: 0.5 Hz, 1.0 Hz (the rate used in the main text), and 2.0 Hz. The history length is held fixed at L=4 in frame units and the CSSR significance level at α=0.001, so that the algorithmic configuration is identical across rates. The movement threshold is specified in pixels/s and converted to the appropriate per-frame value at each rate, so that $Y_{t}$ corresponds to the same physical speed criterion throughout. All other choices—colonies KA and KB, the 2–5 h recording window, and the input symbol $X_{t}$ encoding the change in neighbour count—are unchanged.

Table A2 reports, for each rate, the mean one-step prediction accuracy and the number of inferred causal states for the three model classes. The qualitative pattern reported in the main text is preserved at every rate: the memoryful ϵ-transducer that incorporates the social input *X* yields only a marginal improvement over the memory-only ϵ-machine (a gap of approximately 0.001 in prediction accuracy at every rate), while both memory-based models substantially outperform the memoryless model that uses only the instantaneous input.

The number of causal states of the memoryful transducer grows with the sampling rate. The most parsimonious explanation is a sample-size effect: at a fixed recording window and fixed α, a higher sampling rate yields proportionally more past–present pairs and therefore greater statistical power to retain distinct causal states. The memoryless model’s state count, in contrast, remains constant at 16 across all rates, so the growth of memoryful states does not reflect a stronger dependence on social input.

The corresponding distributions of transition probabilities are shown in Figure A2: the high-probability mode near p≈0.9 accompanied by a broader stochastic component is preserved across all rates for both memory-based models, while the memoryless transducer remains concentrated around p≈0.4–0.6 throughout.

This robustness applies to predictive redundancy within the present encoding and does not bear on the causal-vs-predictive distinction discussed in Section 4.

**Table A2:** Cross-sampling-rate comparison of model performance. Same dataset and parameters as the main analysis (colonies KA and KB, 2–5 h recording window, history length L=4, CSSR significance level α=0.001).

Model	0.5 Hz	1.0 Hz	2.0 Hz
*Mean prediction accuracy*			
Memory-only	0.8126	0.8027	0.7874
Memoryful	0.8136	0.8037	0.7887
Memoryless	0.6123	0.5686	0.5315
*Number of causal states*			
Memory-only	14	13	15
Memoryful	128	152	164
Memoryless	16	16	16

## Appendix C. Robustness to the Movement-Binarisation Threshold

In the main analysis, the binary output $Y_{t}$ is defined by a movement threshold of zero (Section 2.3): any detectable displacement is coded as moving (Y=1) and only a perfectly stationary step as inactive (Y=0). A natural question is whether the reconstructed structure depends on this particular choice. To test this, we repeated the three-model reconstruction (memory-only ϵ-machine, memoryful ϵ-transducer, memoryless ϵ-transducer) at three progressively stricter thresholds, coding a step as moving only when KE>1,5, and 10. Here, the kinetic energy is defined as the square of the instantaneous speed, $\mathrm{KE}=v^{2}$ (in pixels^2^/s^2^), so that the threshold-zero criterion of the main text ($v_{\mathrm{thr}}=0$) is the limiting case KE>0. All other settings are identical to the main analysis.

The resulting transition-probability distributions are shown in Figure A3, and model complexity and one-step prediction accuracy are summarised in Table A3.

Two conclusions hold across all thresholds. First, the predictive redundancy of social input is preserved: at every threshold, the memoryful ϵ-transducer matches the memory-only ϵ-machine in one-step accuracy, despite requiring an order of magnitude more states, while the memoryless transducer remains markedly worse. The deterministic–stochastic structure is likewise retained: the memory-based distributions (memory-only and memoryful) each combine a dominant high-probability (near-deterministic) mode with a broad lower-probability (stochastic) component, as in the main analysis. Note that the absolute accuracy rises with the threshold simply because a growing fraction of steps are coded as (easily predicted) stationary; the relevant invariant is the memoryful–memory-only gap, which remains ≈0.001 at every threshold.

Second, as the threshold increases, the entire distribution shifts toward higher transition probabilities, and the memoryful transducer requires progressively fewer states at the stricter thresholds (KE>1,5,10). This is the expected consequence of removing fine-scale fluctuations: the low-amplitude, intermittent micro-movements within clusters, which generate the most stochastic (p≈0.5) transitions, are gradually reclassified as stationary, so the residual dynamics appear more deterministic and need fewer causal states. Crucially, this is a gradual shift rather than a qualitative change. The stochastic component does not vanish, and the deterministic/stochastic distinction between exploratory and clustering dynamics persists unless the threshold is raised to extreme values that discard most movement. The threshold-zero encoding used in the main text is thus the most inclusive choice—it retains all detectable movement—and the central findings (the redundancy of instantaneous social input and the deterministic–stochastic dichotomy) are not artefacts of where the movement threshold is placed.

**Table A3:** Model complexity and one-step prediction accuracy across movement-binarisation thresholds. KE>0 is the main analysis (Table 1); all other settings are identical.

KE Threshold	Model	States	Accuracy
>0 (same as main text)	Memory-only	13	0.8027
	Memoryful	152	0.8037
	Memoryless	16	0.5686
>1	Memory-only	16	0.8663
	Memoryful	193	0.8671
	Memoryless	16	0.6302
>5	Memory-only	16	0.9163
	Memoryful	145	0.9180
	Memoryless	15	0.7618
>10	Memory-only	16	0.9339
	Memoryful	123	0.9354
	Memoryless	11	0.7979

## Appendix D. Speed–Determinism Relationship Across Colonies and Recording Windows

In the main text (Figure 11), the relationship between instantaneous speed and the determinism of the transitions is shown for colony KA over the 2–3 h window, using the universal ϵ-transducer. Figure A4 reproduces this plot for both analysed colonies (KA, KB) across all three one-hour windows (2–3, 3–4, 4–5 h). The same pattern holds throughout: stochastic transitions are confined to low speed, while high-speed movement concentrates on the near-deterministic self-recursive state. The speed–determinism correspondence is therefore a robust feature across colonies and recording intervals rather than a property of any single recording.

## Appendix E. Visualisation of Reconstructed *ϵ*-Transducer/Machine

In this appendix, we present examples of the reconstructed ϵ-machine and ϵ-transducer obtained from the data. In all figures below, each circle denotes a causal state *s*, and each arrow is a transition labelled Y∣X:p, where *Y* is the output, *X* the input, and p=P(Y∣s,X) the transition probability. Note that the ϵ-machine ignores the input, so X=0 for all arrows in Figure A6.
